# SORBS1 inhibits epithelial to mesenchymal transition (EMT) of breast cancer cells by regulating PI3K/AKT signaling and macrophage phenotypic polarization

**DOI:** 10.18632/aging.205632

**Published:** 2024-03-06

**Authors:** Kai Feng, Ya Di, Meng Han, Weitao Yan, Yimin Wang

**Affiliations:** 1 Department of Surgical, Hebei Medical University, Shijiazhuang 050011, Hebei, China; 2 Department of Oncology, The First Hospital of Qinhuangdao, Qinhuangdao 066600, Hebei, China; 3 Breast Surgery, The First Hospital of Qinhuangdao, Qinhuangdao 066600, Hebei, China; 4 Department of General Surgery, The First Hospital of Qinhuangdao, Qinhuangdao 066600, Hebei, China

**Keywords:** breast cancer cells, PI3K/AKT signaling pathway, macrophage polarization, epithelial mesenchymal transition, SORBS1

## Abstract

This study aimed to explore the regulatory role of SORBS1 in macrophage polarization and the PI3K/AKT signaling pathway, as well as analyze its mechanism in epithelial-mesenchymal transition (EMT) of breast cancer cells. We established SORBS1-overexpressing and knockout cell lines and verified the effects of SORBS1 on cell viability, invasion, and migration by phenotyping experiments and assaying the expression of associated proteins. Furthermore, we established a breast cancer cell and macrophage co-culture system to validate the effect of SORBS1 expression on macrophage polarization and killing of breast cancer cells. Bioinformatics analysis showed that SORBS1 was lowly expressed in breast cancer (BRCA) samples and highly expressed in healthy tissues. Decreased SORBS1 expression was associated with poor prognosis, and the PI3K/AKT signaling pathway was the most significantly enriched pathway. *In vitro* experiments showed that high expression of SORBS1 inhibited the migration of breast cancer cells, as well as the PI3K/AKT signaling pathway, and blocked EMT of these cells. In addition, SORBS1 induced macrophage polarization to the M1-type and enhanced the killing effect on breast cancer cells in the co-culture system. In conclusion, we successfully verified that SORBS1 inhibits the invasion and migration of breast cancer cells, induces macrophage M1-type polarization, and blocks EMT of breast cancer cells, and it may act by regulating the PI3K/AKT signaling pathway.

## INTRODUCTION

According to recent data, the incidence of breast cancer has increased over the past ten years and is still the most common cancer among women, particularly in Asia and Africa, where the rates of both incidence and mortality are still high. It is predicted that by 2040, there will be one million breast cancer-related deaths and over three million new cases [1, 2]. Breast cancer is highly invasive, has a poor prognosis and is difficult to cure [3]. Currently, the commonly used clinical treatments include immunotherapy, targeted therapy, minimally invasive therapy, radiotherapy, and chemotherapy [4, 5]. Advances in medical technology has led to considerable improvement in therapeutics; nevertheless, these medicines still have side effects and are not effective in treating metastatic breast cancer, which has a severe negative impact on the physical and mental health of patients worldwide [6, 7]. To develop more effective therapeutic options for patients with breast cancer, it is imperative to identify novel pharmacological targets.

Sorbin and SH3 domain-containing protein 1 (SORBS1, also known as CAP/ponsin) is a novel transcription factor which regulates cell invasion and proliferation, and restores sensitivity to chemotherapeutic agents [8, 9]. Our biochemical results showed that SORBS1 was abundantly expressed in normal breast tissues, while its expression levels were significantly reduced in breast cancer tissues, and loss of SORBS1 may be closely related to the occurrence and development of breast cancer. It was found that miR-142-5p promotes breast cancer cell proliferation, invasion, and migration by targeting SORBS1 [10]. As a potential bioindicator of breast cancer, SORBS1 is very valuable. Sheng-Hua Li et al. demonstrated that there was a positive correlation (p<0.001) between the mRNA expression levels of SORBS1 and macrophages in bladder cancer [11]. Functional enrichment analysis revealed that the epithelial-mesenchymal transition route was the most enriched pathway for genes associated with SORBS1, indicating a potential relationship between SORBS1, EMT and macrophages.

The PI3K/AKT signaling pathway, one of the most important signaling pathways involved in regulating EMT, was found to be the most significantly enriched pathway as revealed by functional enrichment analysis. This pathway activates E-cadherin and decreases N-cadherin expression, thereby promoting the development of EMT [12, 13]. The proliferation and invasiveness of breast cancer cells can be decreased, which lowers the risk of breast cancer development, by blocking the activation of the PI3K/AKT signaling pathway [14, 15]. In addition, Haoran Shen et al. found that SORBS1 is associated with the phosphorylation of PI3K and AKT, and plays an important role in the pathogenesis of polycystic ovary syndrome [16]. Nevertheless, the relationship between EMT, macrophage polarization, SORBS1, and the PI3K/AKT signaling pathway in breast cancer has not yet been elucidated. Therefore, the present study focused on the regulation of the PI3K/AKT signaling pathway and macrophage polarization by SORBS1 to inhibit EMT in breast cancer, with the aim of discovering potential drug targets for the clinical treatment of this disease.

## MATERIALS AND METHODS

### Data collection and processing

We gathered mRNA expression profiles and relevant clinical information of patients with breast cancer from GEO (https://www.ncbi.nlm.nih.gov/geo/) and TCGA (https://portal.gdc.cancer.gov/). The GSE10810 and GSE42568 datasets were downloaded from the GEO database. Differentially expressed genes (DEGs) in GSE10810 and GSE42568 were identified using GEO2R (https://www.ncbi.nlm.nih.gov/geo/geo2r/), a tool designed for GEO online analysis using the R programming language. DEGs in both of the datasets were identified according to the adjusted p-value < 0.05 and |logFC| ≥ 2 as criteria. We downloaded the RNA sequencing data in transcripts per million (TPM) format from the UCSC Xena database (http://ualcan.path.uab.edu/) for pan-cancer analysis. The protein expression levels of SORBS1 were assessed using the UALCAN database (http://ualcan.path.uab.edu/) [17].

### Clinical sample collection

All breast tissue samples were procured from the Department of Pathology, Qinhuangdao First Hospital. Clinicopathological information of the patients was collected from the hospital medical record system, and informed consent of patients was obtained for all human samples. None of the patients received neoadjuvant chemotherapy, radiation therapy, or any other specialized care before surgery. The hospital’s ethics committee approved the use of samples and data for the study.

### Real-time quantitative PCR

Twenty pairs of primary breast cancer (BRCA) samples and adjacent normal tissues were collected at the Qinhuangdao First Hospital. Total RNA was extracted from the tissues using TRIzol reagent following the manufacturer’s instructions (Solarbio, Beijing, China). Complementary DNA was synthesized using the Hifair II 1st Strand cDNA Synthesis SuperMix (Yeasen, Shanghai, China), and the product was used for reverse transcription. Next, qPCR was performed using Hieff qPCR SYBR Green Master Mix (Yeasen, Shanghai, China) on a LightCycler 480 Instrument II (Roche, Basel, Switzerland). The analyses were performed using GraphPad Prism 10.0.1 software. qPCR primer sequences and si-SORBS1 sequences are listed in Supplementary Table 4, and the SORBS1-overexpressing plasmid construct is shown in Supplementary Figure 1. The company, RiboBio (Guangzhou, China) constructed and synthesized the overexpression plasmids and siRNA.

### Immunohistochemistry

In the current study, 20 breast cancer and paracancerous tissues were examined by immunohistochemistry. The tissues were collected, embedded in paraffin, and sliced into 6 mm slices after being fixed in 4% paraformaldehyde. The slices were treated with 33% H_2_O_2_ and EDTA (pH 9.0) for complete antigen retrieval. Then, the slices were rinsed with PBS (pH 7.4) and incubated with SORBS1 antibody (1:500; Abcam, USA) for 1 h at 37° C. The slices were then incubated with the rabbit anti-rabbit IgG secondary antibody (1:200; SPA237, Solarbio, China), and the positive sites were marked with DAB (diaminin).

### Cell culture

Breast cancer cell lines MCF-7, MDA-MB-453, MDA-MB-231, and SKBR3, normal mammary epithelial cell line MCF 10A, and the human macrophage cell line THP-1 were purchased from the Cell Bank of the Chinese Academy of Sciences, Shanghai, China. Cells were cultured in RPMI 1640 medium (11995, Gibco, USA) containing 10% fetal bovine serum (YSN0121, ExCell Bio, China) and 1% double antibiotics (penicillin-streptomycin mixture) (P1400, Solarbio), and the cells were cultured in an incubator (Jiemei Electronics, China, CI-, at 37° C with 5% CO_2_, 191C). The medium was changed every two days and the cells were cultured until the exponential phase for subsequent experiments.

### Cell transfection

MDA-MB-231 and SKBR3 breast cancer cells in the exponential growth phase were trypsinized, and the cells were centrifuged and resuspended, and then 1 mL of cell suspension (3×10^5^ cells/mL) was added to each well of 6-well plates. After adding 1 mL of cell suspension to each well, 1 mL of RPMI 1640 medium was replenished, and the cells were cultured overnight. Next day, the cells were transfected with either siRNA or overexpression plasmid using Lip 3000 transfection reagent (L3000075, Thermo Fisher Scientific, USA). After 48 h, the transfected cells were used in subsequent experiments.

### CCK8 assay

MDA-MB-231 and SKBR3 breast cancer cell lines were successfully transfected, and the cells were resuspended and plated in 96-well plates at 3×10^4^ cells/mL. MCF-7 breast cancer cell suspension (100 μL) was added to each well, supplemented with 100 μL of RPMI 1640 medium. Incubation was continued in a cell culture incubator for 0, 24, 48, and 72 h. After incubation, 20 μL of CCK8 solution was added to each well (C0038, Beyotime Biotechnology, China) and placed in the incubator for 3 h. The cells were removed, placed in enzyme markers, and the absorbance was measured at 450 nm. Cell viability calculation formula = (OD value of experimental group - OD value of blank control group)/(OD value of control group - OD value of blank control group).

### Colony-forming assay

The MDA-MB-231 and SKBR3 breast cancer cell lines, that were successfully transfected, and 2 mL of cell suspension (3×10^2^ cells/mL) were added to each well of 6-well plates. The cells were incubated for 14 days, and fresh medium was added every 3 days. At the end of the culture period, tissue cell fixative was added, and the cells were fixed for 30 min, and subsequently stained with 0.5% crystal violet for 5 min. The cells were then washed with PBS solution to remove excess crystal violet, dried naturally, and analyzed for the number of colony-forming cells using ImageJ.

### Cell scratch assay

After 48 h of transfection, the cells were incubated in a 6-well plate until the cells reached 95%-100% confluency. The medium was replaced with serum-free RPMI 1640 medium, and a sterile pipette tip was used to draw three horizontal lines on the cell monolayer. The detached cells were washed with PBS, and the picture of the scratch wound was taken. The cells were then incubated for 12 h, and then, the rate of wound healing was quantitated using ImageJ.

### Transwell assay

Cell suspension (100 μL, 1×10^6^ cells/mL), was added to the upper chamber of the glue spreading chamber, placed in the incubator, and incubated for 12 h. The cells were washed thrice with PBS and 500 μL of methanol solution was added into each well of the lower chamber, and incubated for 25 min. Crystal violet solution (0.5%) was added to each well of the lower chamber to stain the cells for 5 min, and the excess crystal violet was washed away with PBS. The cells in the upper chamber were wiped with a cotton swab, dried naturally, and five fields of view were randomly selected to be photographed under a microscope. The images were statistically analyzed using ImageJ software to determine the number of cells with invasive ability.

### Induction of THP-1 cell differentiation to macrophages

THP-1 cells in the exponential phase were plated in 6-well plates at a density of 7×10^5^ cells/well, followed by stimulation with 200 ng/mL phorbol ester (PMA) for 72 h to allow THP-1 cells to differentiate into macrophages for subsequent experiments.

### Co-culture of breast cancer cells with macrophages

For CCK8 assay, macrophages were transfected with SORBS1 siRNA or overexpression plasmid for 48 h. Macrophages were collected and plated in 96-well plates at a density of 3×10^3^ cells/well, and an equal number of breast cancer cells were added; the plates were incubated for 48 h. Excess macrophages were washed away with PBS, and 200 μL of RPMI 1640 medium (containing 10% CCK8) was added to each well and incubated for 3 h. The subsequent assays were performed as described previously. For the transwell experiment, macrophages were transfected with SORBS1 siRNA or overexpression plasmid for 48 h. Macrophages were collected and plated in the upper chamber of the transwell at a density of 1×10^5^ cells/well. An equal number of breast cancer cells were added, and the cells were incubated overnight, followed by fixation and staining, as described previously. For Western blotting and qPCR experiments, after THP-1 cells were induced into macrophages, cells were collected after 48 h of transfection with SORBS1 siRNA or overexpression plasmid, and macrophage proteins and mRNA were extracted for subsequent experiments.

### Western blotting

After MDA-MB-231 and SKBR3 cells were transfected for 48 h, cells were collected by scraping into 1.5 mL centrifuge tubes and set aside. Cell lysis solution (100 μL) (Solarbio, R0010) containing 1% protease inhibitor (Solarbio, P0100) and 1% protein phosphatase inhibitor (Solarbio, P1260) was added and the cells were lysed for 30 min, and centrifuged. The supernatant was collected and the protein concentration of the samples was measured using the BCA Protein Quantification Kit (Solarbio, PC0020). The protein concentration of each group was adjusted to be the same, and the samples were heated in a 95° C water bath for 15 min, and the protein samples were then subjected to SDS-PAGE. The initial voltage was adjusted to 80 V, and subsequently modulated to 120 V after 30 min, and the duration of electrophoresis was 60 min. The proteins were then transferred onto the PVDF membrane, blocked using 5% skim milk powder for 2 h, and incubated with the corresponding primary antibody at 4° C overnight. The membrane was then incubated with the secondary antibody, prepared using 5% skim milk powder, for 1 h. After incubation, the membrane was washed three times with 1× TBST solution for 30 min each time, chemical development solution was added dropwise, and imaged in a gel imager, and the images were used for subsequent data processing and analysis using ImageJ. SORBS1 (ab4551, Abcam, USA), E-Cadherin (Bioss, bs-1519R, China), N-Cadherin (ABclonal, A19083, China), vimentin (ABclonal, A19607, China), GAPDH (CST, 5174S, USA), AKT (Affinity, AF0836, China), p-AKT (Huaan Biologicals, ET1609-51, China), p-AKT (Huaan Biologicals, ET1607-73, China), p-PI3K (Affinity, AF3241, China), PI3K (Affinity, AF6241, China), goat anti-rabbit (Affinity, S0001, China), goat anti-mouse (Affinity, S0002, China).

### Flow cytometry

The transfected THP-1 cells were incubated with cell fixative for 30 min, primary antibody was added and incubated at room temperature for 2 h. The cells were incubated with fluorescent secondary antibody of Euromonitor for 30 min, and detected by flow cytometry, after which the data were analyzed by Flow Jo 10.8.1 software. CD68 (ER1901-32), CD86 (ET1606-50), CD163 (ET1704-43), CD206 (ET1702-04), iFluor™ 488-conjugated goat anti-rabbit IgG goat polyclonal antibody (HA1121) were purchased from Huabio Technologies Co., Ltd., China.

### Functional enrichment analysis

The 32 TCGA cancer-associated multidimensional datasets were analyzed using the LinkedOmics database (http://www.linkedomics.org/). Differentially expressed genes in TCGA BRCA cohort related to SORBS1 were identified using the LinkFinder module. The relevance of the outcomes was analyzed using Pearson’s correlation coefficient, as shown in the volcano plots and heatmaps. To accurately reflect the function of SORBS1, the top 300 genes that were most strongly correlated with SORBS1were identified and chosen for enrichment analysis. KEGG pathway data were analyzed using the “ClusterProfiler” package and the results were visualized using the “ggplot2” package.

### Immune infiltration analysis

The degree of immune infiltration was measured using 24 types of immune cells, and the relative enrichment score of these immune cells in the BRCA cohort was evaluated using ssGSEA using the R package GSVA. The Wilcoxon rank-sum test was used to compare the levels of immune infiltration between the high and low SORBS1 expression groups, and Spearman’s correlation analysis was performed to examine the relationship between SORBS1 and these immune cells.

### Statistical analysis

All statistical analyses were performed using the R software (version 3.6.3) (https://www.r-project.org/). The statistical significance of SORBS1 expression in the paired sample t-test was used to evaluate the paired data, and unpaired tissues were assessed using the Wilcoxon rank-sum test. The relationship between clinical characteristics and SORBS1 expression was evaluated using the Wilcoxon rank-sum test. This was performed using receiver operating characteristic (ROC) curve analysis, and area under the curve (AUC) was used as an indicator of diagnostic precision. The median expression level of SORBS1 served as the cut-off value for the Kaplan-Meier analysis, and the log-rank test was employed for survival analysis. Cox regression analyses using univariate and multivariate variables were performed to determine the effect of clinical factors on patient outcomes. Statistically significant variables were identified using univariate Cox regression analysis and these were further analyzed using multivariate Cox regression. The forest map was generated using the ggplot2 R package. All statistical inferences were two-sided, p-values below 0.05 were considered statistically significant.

### Availability of data and materials

The data in this article are from The Cancer Genome Atlas (TCGA) and the Gene Expression Omnibus (GEO). Raw data can be downloaded from this website: https://www.cancer.gov/about-nci/organization/ccg/research/structural-genomics/tcga; https://www.ncbi.nlm.nih.gov/geo/download/?acc=GSE10810; https://www.ncbi.nlm.nih.gov/geo/download/?acc=GSE42568.

### Consent for publication

All authors and participants agreed to publish this paper.

## RESULTS

### SORBS1 expression analysis

The cohort of this study included relevant clinical and pathological information and RNA sequencing data from 1,109 breast cancer patients. Data for 113 cancer tissue samples along with paired adjacent normal breast tissues were obtained from TCGA. The clinicopathological features of patients with BRCA are shown in Supplementary Table 1.

**Figure 1:**
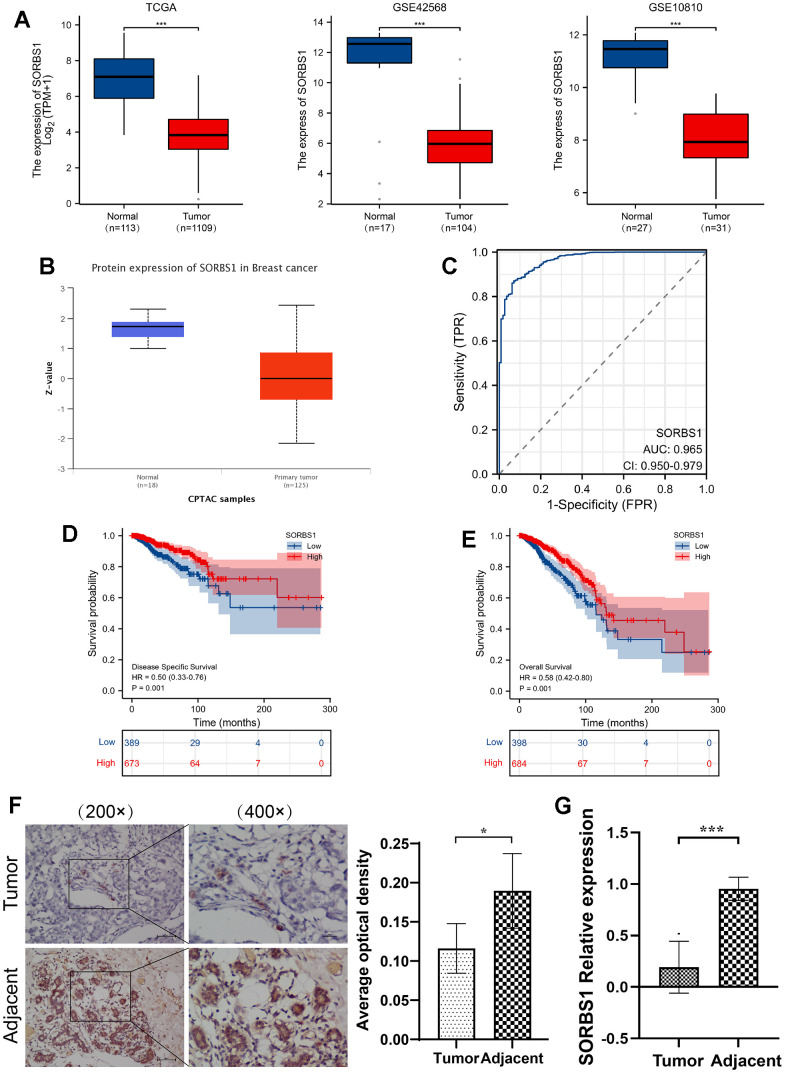
**Relative expression of SORBS1 in breast cancer based on raw letter analysis.** (**A**) The expression of SORBS1 mRNA in breast cancer and non-matched normal tissues based on the TCGA database, and the expression of SORBS1 mRNA in breast cancer tissues and adjacent tissues based on the GEO database. (**B**) The protein expression level of SORBS1 is based on CPTAC. (**C**) ROC curves of breast cancer and normal breast tissue classified in TCGA database. (**D**, **E**) Prognostic values of SORBS1 expression in patients with breast cancer evaluated by the Kaplan-Meier method. Overall survival and disease-specific survival (**B**) for breast cancer patients with high versus low SORBS1. (**F**) Representative images of SORBS1 protein expression in BRCA tissues and adjacent normal tissues. Original magnifications 200× and 400×. (**G**) Quantitative real-time PCR analysis of BRCA tissues and adjacent normal tissues. (^***^ *P*<0.001).

The pan-cancer study revealed that SORBS1 expression was generally low in all cancer types (Supplementary Figure 1A). Compared to normal samples, SORBS1 expression was markedly reduced in BRCA. Additionally, we retrieved the microarray datasets from the GEO database, namely, GSE42568 and GSE10810, to confirm the results mentioned above, which showed that SORBS1 expression was generally low in all cancer types. Moreover, the microarray data from these datasets showed that breast cancer tissues had much lower levels of SORBS1 mRNA expression than breast tissues in healthy subjects. (p<0.001, Figure 1A). The datasets presented a similar trend at the protein level (p<0.001 Figure 1B). Additionally, the ROC curve revealed that SORBS1 showed a high predictive ability between breast cancer and normal tissues, with AUC of 0.965 (95% confidence interval [CI]), confidence interval ([CI]) = 0.951-0.979) (Figure 1C). Using qPCR and immunohistochemistry, we further validated the expression of SORBS1 in clinical BRCA samples and found that SORBS1 expression in BRCA tissues is a significant factor in the development of BRCA. Its expression in BRCA tissues was significantly lower than that in adjacent normal breast tissues (Figure 1F, 1G).

### Prognostic value of SORBS1 in breast cancer

We used the Kaplan-Meier method to analyze the relationship between SORBS1 expression and the chances of surviving breast cancer. We divided the patients into groups with high and low SORBS1 expression, using the median value of SORBS1 expression as the cut-off score. The OS and DSS of the low SORBS1 expression group were significantly poorer than those of the high SORBS1 expression group, indicating significantly poorer prognosis than the high SORBS1 expression group (OS: hazard ratio [HR] = 0.58, 95% CI= 0.42 -0.80, p<0.001; DSS: HR = 0.50, 95% CI= 0.33-0.76, p< 0.001) (Figure 1D, 1E). Univariate and multivariate regression models were used to determine whether SORBS1 is a standalone prognostic factor for breast cancer. Multivariate analysis identified SORBS1 expression, M stage, age, pathological stage, and ER status as independent predictors of OS. Likewise, SORBS1 expression, M stage, and pathological stage were demonstrated to be prognostic markers for DSS (Supplementary Figure 1C, 1E and Supplementary Tables 2, 3).

### Correlation between SORBS1 expression and immune infiltration

**Figure 2:**
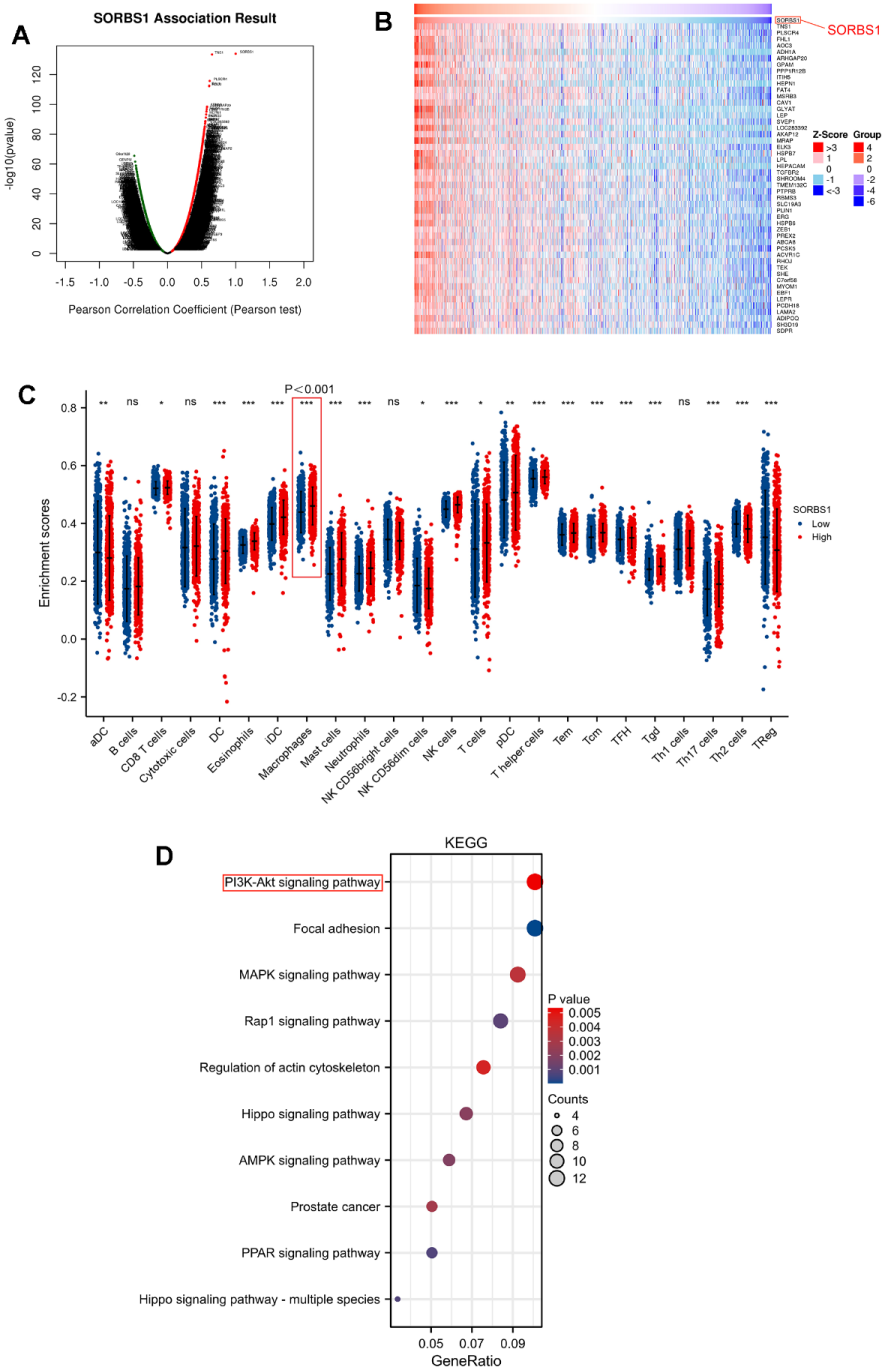
**Enrichment analysis of SORBS1 functional network and immune cell ratio analysis in BRCA.** (^*^ p<0.05, ^**^ p<0.01, ^***^ p<0.001, ^****^ p<0.0001). (**A**) Genes highly related to SORBS1 were identified in the BRCA cohort by Pearson test. (**B**) The heat map shows the top 50 genes positively related to SORBS1 in the BRCA cohort. (**C**) The varied proportions of 24 types of immune cells in high and low SORBS1 expression groups in tumor samples. (**D**) Enrichment of KEGG terms for genes related to SORBS1.

Correlation analysis between SORBS1 expression and infiltrated immune cells revealed that SORBS1 expression was positively correlated with 13 types of infiltrated immune cells, including Tcm (r = 0.375, p< 0.001), mast cells (r = 0.370), NK cells (r = 0.354), eosinophils (r = 0.318), iDC (r = 0.257), neutrophils (r = 0.222), T helper cells (r = 0.206), Tgd (r = 0.191), macrophages (r = 0.100), Tem (r = 0.153), Th17 cells (r = 0.125), TFH (r = 0.117) and DC (r = 0.114), while correlation between r and TFH is not significant (0.114). Its expression was negatively correlated with the infiltration of Th2 (r = -0.224), TReg (r = -0.172), aDC (r = -0.117), and NK CD56 dim cells (r = -0.112) in BRCA (Figure 2C and Supplementary Figure 1F).

### SORBS1 co-expression network in BRCA

The LinkFinder module in the LinkedOmics web portal was used to investigate the co-expression pattern of SORBS1 in TCGA-BRCA and to determine the biological role of SORBS1 in BRCA. Nine thousand genes (dark red) were positively correlated with SORBS1, and 5699 genes (dark blue) were negatively correlated (Figure 2A). The top 50 genes that were negatively and positively related to SORBS1 are depicted in heatmaps (Figure 2B and Supplementary Figure 1C). KEGG pathway analysis indicated enrichment of the PI3K/AKT signaling pathway, regulation of the actin cytoskeleton, ECM-receptor interaction, focal adhesion, and Rap1 signaling pathway, AMPK signaling pathway, and PPAR signaling pathway (Figure 2D).

### SORBS1 inhibits proliferation, invasion and migration of breast cancer MDA-MB-231 and SKBR3 cells

**Figure 3:**
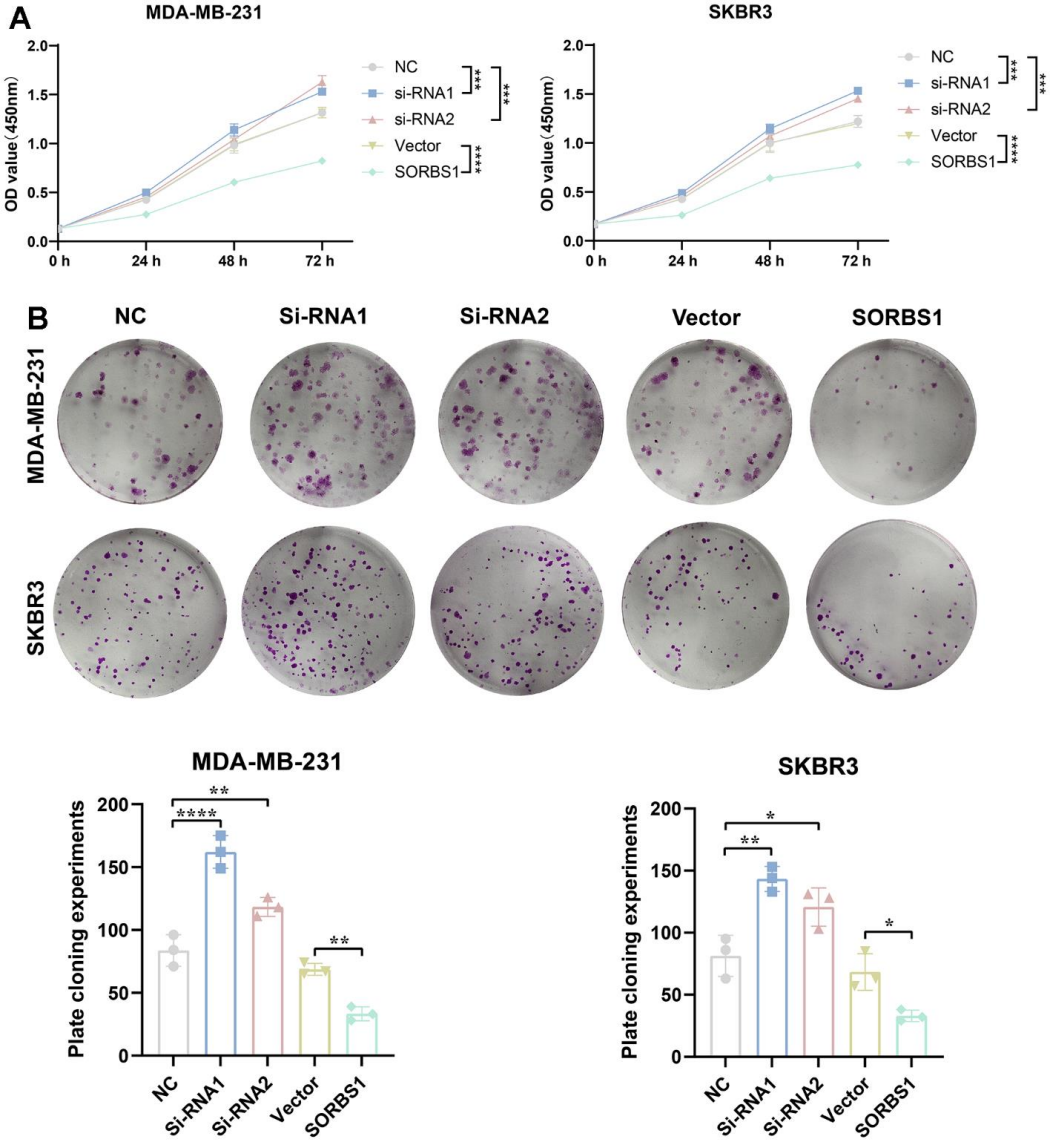
**SORBS1 inhibits proliferation of MDA-MB-231 and SKBR3 cells.** MDA-MB-231 and SKBR3 cells were transfected with SORBS1 overexpression plasmid and siRNA for 48 h. (**A**) Changes in cell viability of MDA-MB-231 and SKBR3 cells were detected by CCK8, and (**B**) changes in proliferative capacity of MDA-MB-231 and SKBR3 cells after 48 h of transfection were detected by clone formation assay (the CCK8 experiment counted the experimental results for 72 h, ^*^ *P* < 0.05, ^**^ *P* < 0.01, ^***^ *P* < 0.001, ^****^ *P* < 0.0001).

**Figure 4:**
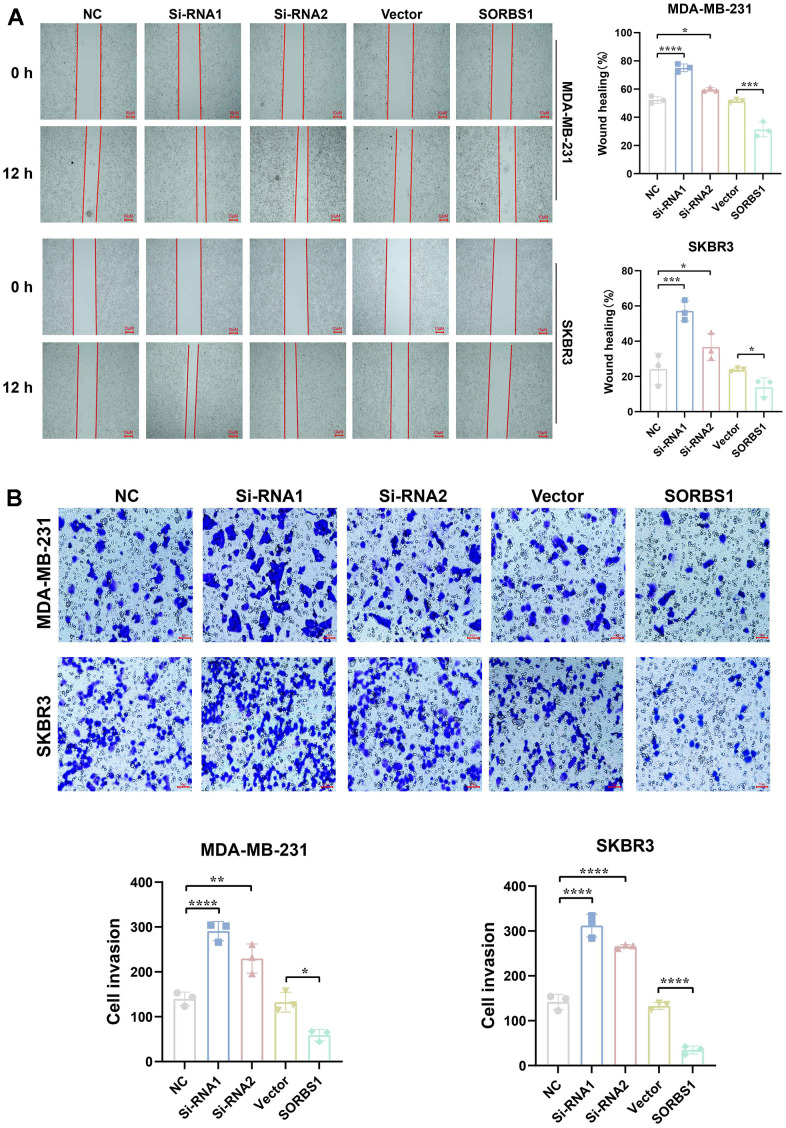
**SORBS1 inhibits MDA-MB-231 and SKBR3 cell invasion and migration.** MDA-MB-231 and SKBR3 cells were transfected with SORBS1 overexpression plasmid and siRNA for 48 h. (**A**) Changes in the migratory ability of MDA-MB-231 and SKBR3 cells were detected by scratch assay. (**B**) Changes in the invasive ability of MDA-MB-231 and SKBR3 cells were detected by transwell assay (^*^ *P* < 0.05, ^**^ *P* < 0.01, ^***^ *P* < 0.001, ^****^ *P* < 0.0001).

CCK8 and colony-forming assays showed that SORBS1 overexpression inhibited the proliferation of MDA-MB-231 and SKBR3 cells, whereas the cell viability of these cells was significantly enhanced after SORBS1 knockdown (Figure 3A, 3B). We subsequently verified the effect of SORBS1 expression on cell invasion and migration by scratch wound and transwell assays, and as expected, overexpression of SORBS1 inhibited MDA-MB-231 and SKBR3 cell invasion and migration (Figure 4A, 4B).

### SORBS1 regulates the PI3K/AKT signaling pathway and inhibits EMT in breast cancer cells

**Figure 5:**
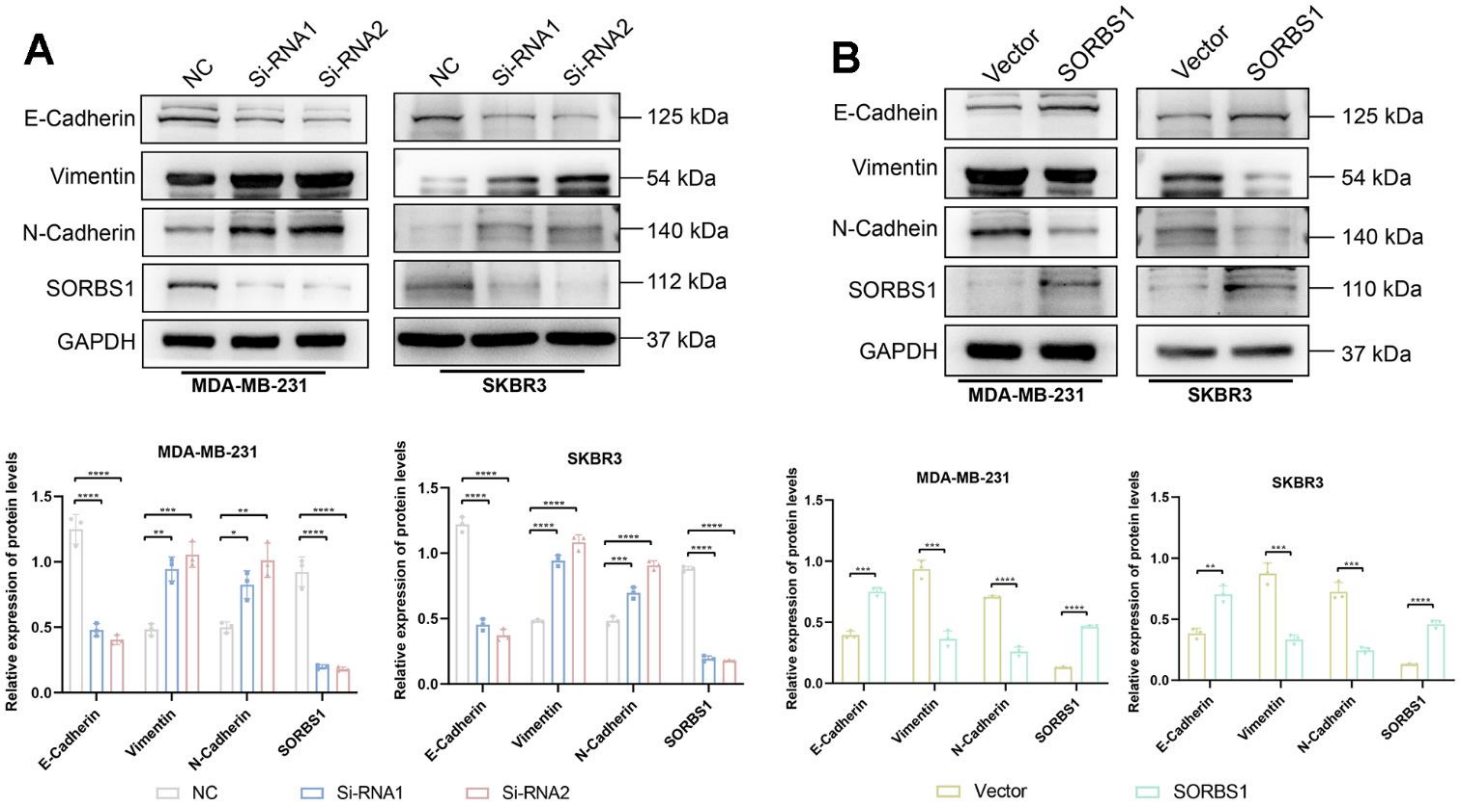
**SORBS1 can regulate EMT-related proteins.** (**A**) The expression of EMT-related proteins was detected 48 h after transfection of MDA-MB-231 cells with siRNA and overexpression plasmid of SORBS1. (**B**) The expression of EMT-related proteins was detected after transfecting SKBR3 cells with siRNA and overexpression plasmid of SORBS1 for 48 h. (^*^ *P* < 0.05, ^**^ *P* < 0.01, ^***^ *P* < 0.001, ^****^ *P* < 0.0001).

**Figure 6:**
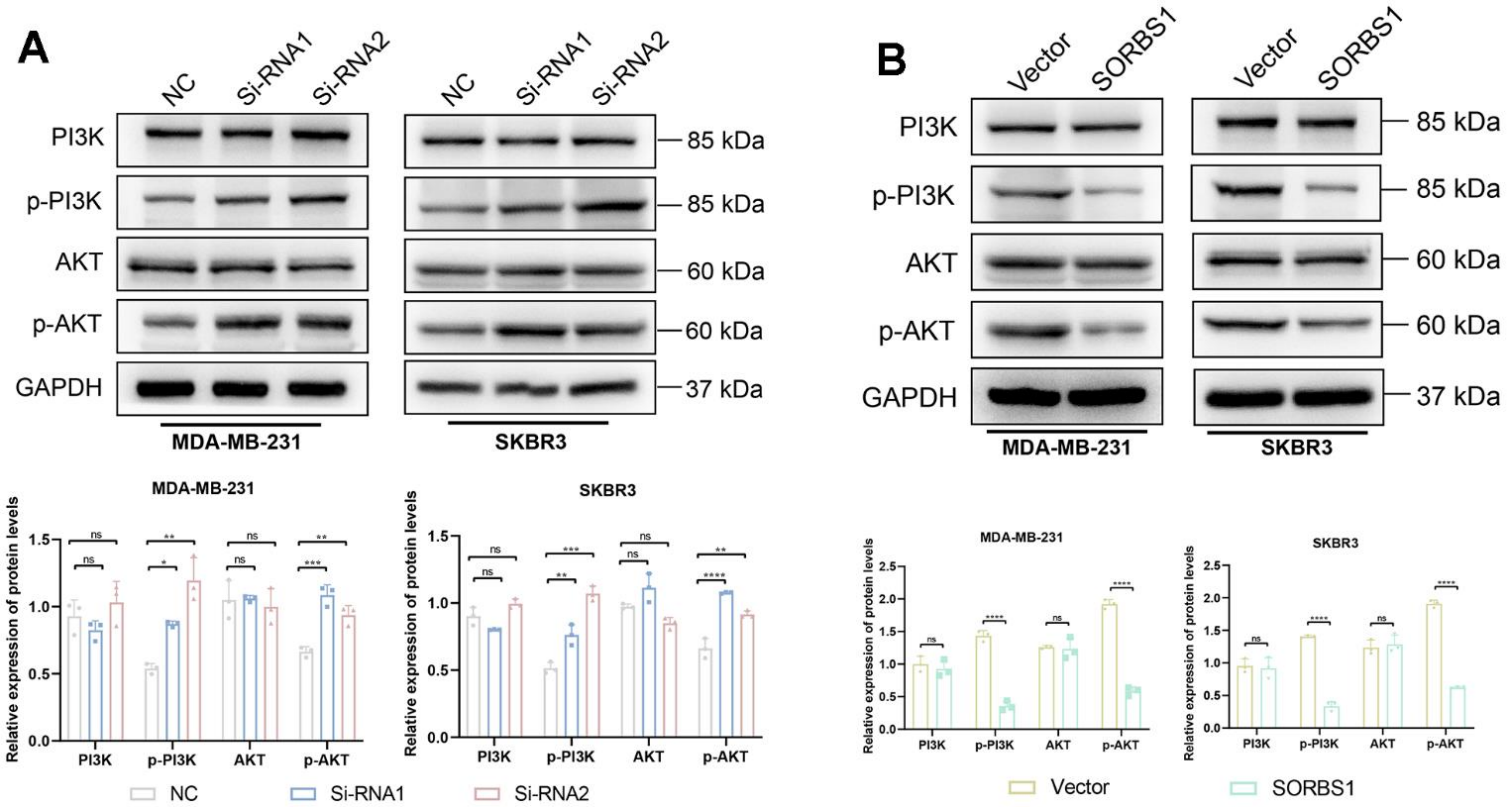
**SORBS1 inhibits the PI3K/AKT signaling pathway.** (**A**) The expression of PI3K, p-PI3K, AKT, and p-AKT proteins was detected by Western blot assay in MDA-MB-231 cells. (**B**) Expression of PI3K, p-PI3K, AKT, p-AKT proteins was detected by Western blot assay in SKBR3 cells. (^*^ *P* < 0.05, ^**^ *P* < 0.01, ^***^ *P* < 0.001, ^****^ *P* < 0.0001).

We detected the changes in the expression of proteins in the PI3K/AKT signaling pathway and EMT-related proteins by Western blotting. In MDA-MB-231 and SKBR3 cells overexpressing SORBS1, the expression levels of p-AKT, p-PI3K, N-cadherin, and vimentin were decreased as compared with the control group. The expression levels of E-cadherin were increased, while the expression of AKT and PI3K remained unchanged. However, SORBS1 knockdown resulted in increased levels of p-AKT, p-PI3K, N-cadherin, and vimentin, and reduced levels of E-cadherin compared to control cells, whereas the expression of AKT and PI3K remained unchanged (Figures 5, 6).

### SORBS1 induces macrophage M1-type polarization to inhibit invasion and metastasis of breast cancer cells

**Figure 7:**
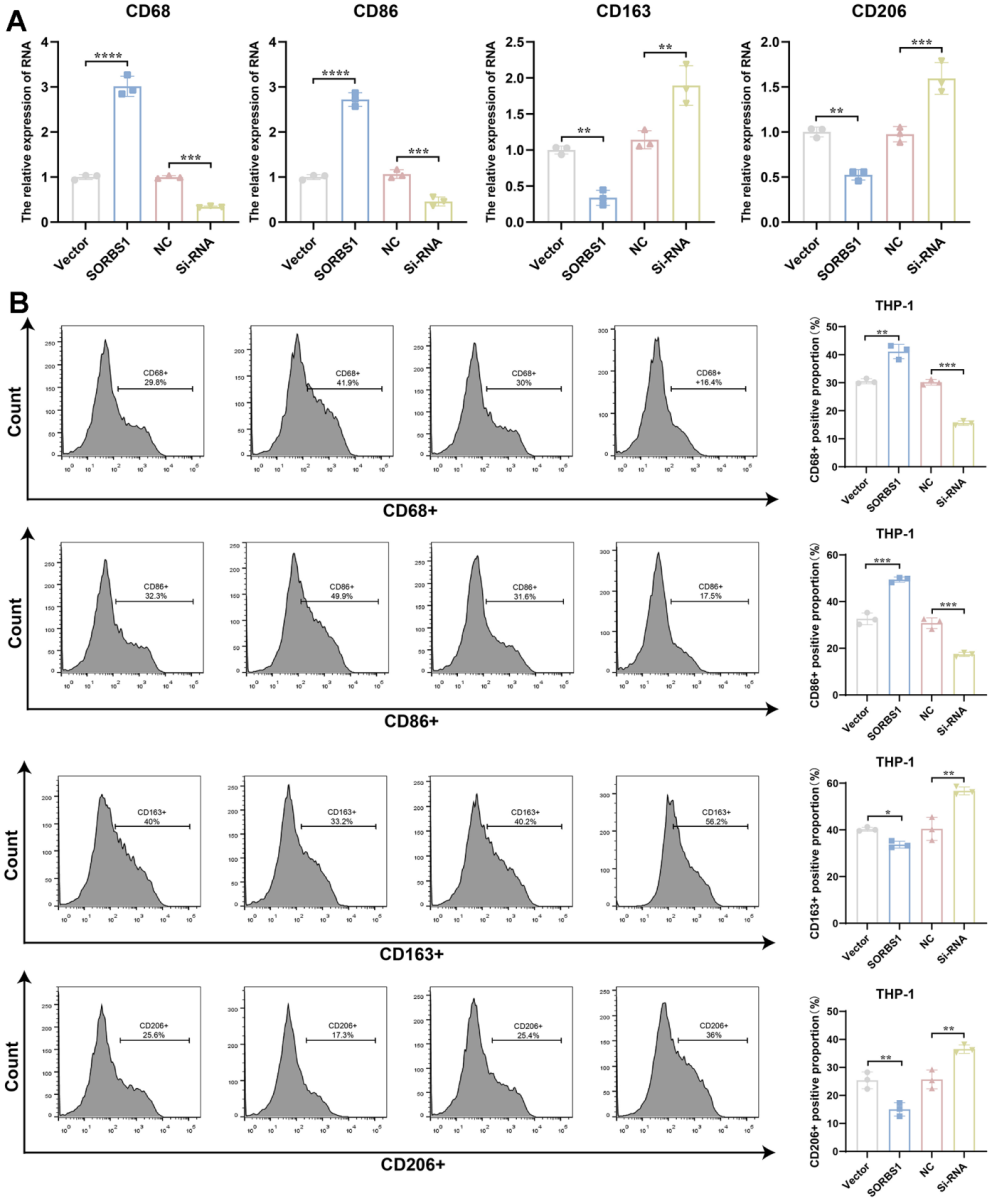
**SORBS1 induces M1-type polarization in THP-1 cells.** After transfection of induced differentiated THP-1 cells with SORBS1 overexpression plasmid and siRNA for 48 h. (**A**) Expression levels of CD68, CD86, CD163, and CD206 mRNAs were detected by QPCR assay. (**B**) Changes in the proportions of CD68+, CD86+, CD206+ and CD163+ were detected by flow assay in THP-1 cells after different treatments. (^*^ *P* < 0.05, ^**^ *P* < 0.01, ^***^ *P* < 0.001, ^****^ *P* < 0.0001).

**Figure 8:**
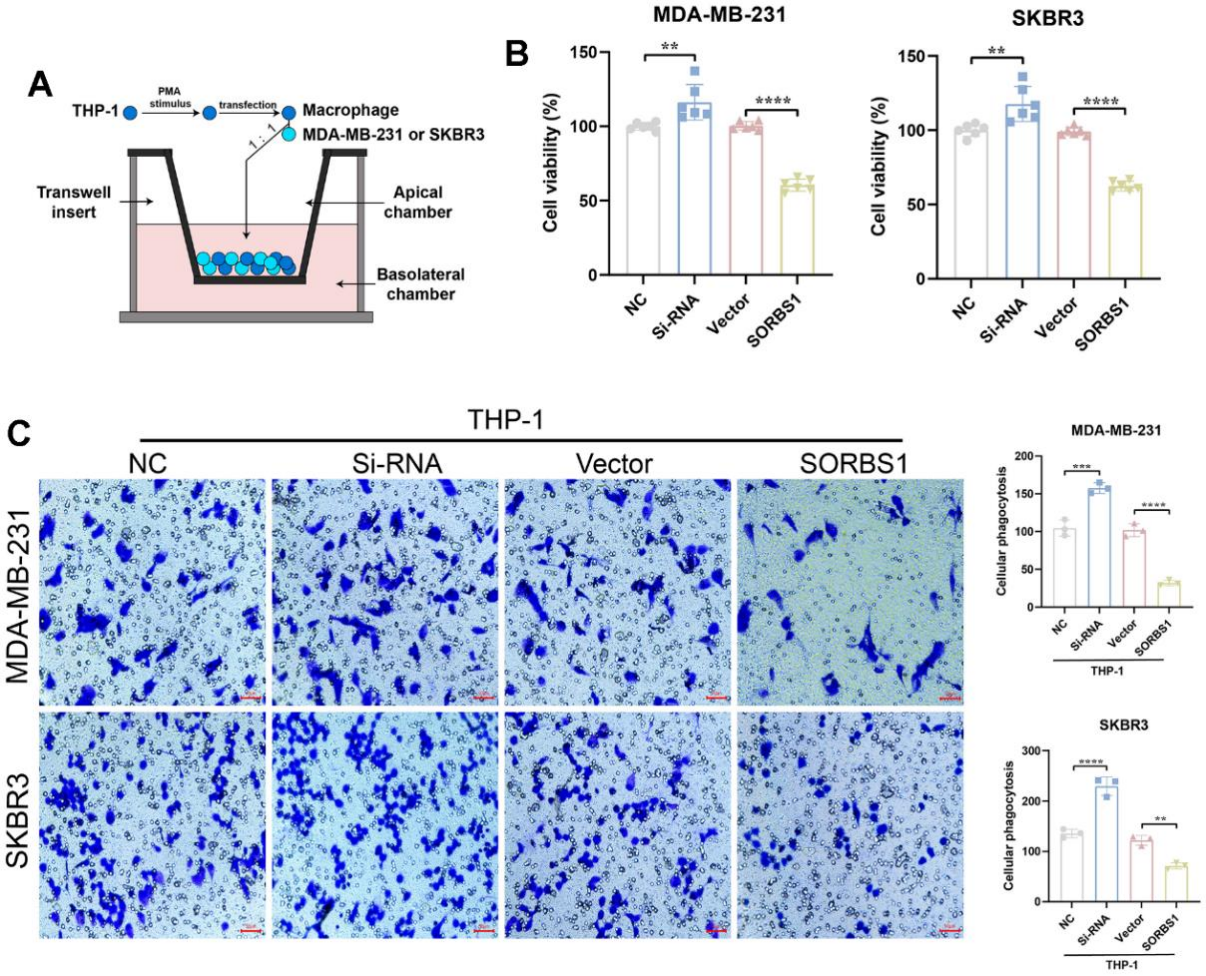
**SORBS1 induces THP-1 polarization to inhibit breast cancer cell proliferation and invasion.** (**A**) Schematic diagram of THP-1 cells co-cultured with SORBS1. (**B**) Changes in the co-culture viability of MDA-MB-231 and SKBR3 cells after 48 h of co-culturing as detected by CCK8 assay. MB-231 and SKBR3 cell viability changes. (**C**) Changes in MDA-MB-231 and SKBR3 cell invasive ability after 48 h of co-culture as detected by transwell assay and by CCK8 assay (^*^ *P* < 0.05, ^**^ *P* < 0.01, ^***^ *P* < 0.001, ^****^ *P* < 0.0001).

The results of qPCR experiments showed that in THP-1 cells differentiated into macrophages, the mRNA expression levels of both CD68 and CD86 were significantly increased after overexpression of SORBS1 compared to the control group, whereas the mRNA expression levels of both CD206 and CD163 were significantly decreased (Figure 7A). Flow cytometry revealed that SORBS1 increased the percentages of CD68+ and CD86+ cells and decreased the percentages of CD206+ and CD163+ cells. In conclusion, SORBS1 induced M1-type polarization in THP-1 cells (Figure 7B). The results of CCK8 and transwell assays showed that, upon co-culture of THP-1 cells overexpressing SORBS1 with either MDA-MB-231 or SKBR3 cells overnight, the invasive ability of MDA-MB-231 and SKBR3 cells was significantly reduced, and the killing effect of THP-1 cells on MDA-MB-231 and SKBR3 cells was significantly enhanced. However, SORBS1 knockdown showed the opposite result upon co-culture of THP-1 cells with breast cancer cells (Figure 8).

## DISCUSSION

The role of SORBS1 in several malignant tumors has been reported in previous studies, including colorectal [18], gastric [19] and ovarian cancers [20]; however, its role in BRCA is unknown. Therefore, it is necessary to clarify the role of SORBS1 in the prognosis, progression, and treatment of breast cancer. In this study, our bioinformatics analysis of SORBS1 showed that its expression was significantly decreased at both the mRNA and protein levels in BRCA tissues compared to that in normal tissues, and our findings are consistent with previous investigations [21]. ROC analysis confirmed the diagnostic utility of SORBS1 in patients with BRCA with high confidence scores. Similarly, many previous studies have demonstrated the importance of SORBS1 as a possible prognostic predictor in patients with various solid malignancies such as metastatic prostate [22], cervical [23] and breast cancers [24]. We performed KEGG analysis of SORBS1-correlated genes using TCGA data and found that the PI3K/AKT signaling pathway was the most significantly enriched signaling pathway. The results of other studies also confirmed the association of SORBS1 with the PI3K/AKT signaling pathway [16]. Subsequently, we confirmed the inhibitory effect of SORBS1 on breast cancer cell proliferation and invasion *in vitro*, which is consistent with the results of Yu et al. [10]. However, the inhibitory mechanism of SORBS1 on breast cancer cells has not been entirely explored by previous researchers. Based on the results of our previous bioinformatics analysis, we focused on the PI3K/AKT signaling pathway, and we demonstrated for the first time that SORBS1 inhibited the activation of p-AKT and p-PI3K, increased the expression levels of E-cadherin, and decreased the expression levels of N-cadherin, thereby indicating the inhibitory effect of SORBS1 on the proliferation and invasion of breast cancer cells. The relationship between SORBS1 and the PI3K/AKT signaling pathway and the regulation of EMT has been preliminarily elaborated, and the ability of the PI3K/AKT signaling pathway to regulate EMT has been confirmed by several experiments conducted by previous researchers [25]. In the literature, it has been reported that the absence of E-cadherin and vimentin protein expression and the aberrant expression of N-cadherin protein led to more invasive breast cancer [26]. Therefore, E-cadherin, N-cadherin and vimentin are key proteins in EMT of breast cancer cells, and our results have confirmed the regulatory role of SORBS1 on E-cadherin, N-cadherin and vimentin, which is an exciting result.

Macrophages are the main infiltrating immune cell type in BRCA-associated triple-negative breast cancer [27]. Macrophages can enhance anti-tumor effects through M1-type polarization, and the combination of macrophage polarization inducers with targeted therapies shows great promise, and some researchers have already counteracted the challenge of clinical drug resistance by inducing macrophage polarization [28]. The present study revealed that SORBS1 induces macrophage polarization. In this study, we found that SORBS1 up-regulated the expression levels of CD68 and CD86 mRNA and down-regulated the expression levels of CD206 and CD163 mRNA, which in turn led to the differentiation of macrophages to the M1-type, enhanced their killing effect on breast cancer cells, and inhibited the invasion of these cells. Our bioinformatics results showed that the expression of SORBS1 was positively correlated with macrophages in BRCA, and Sheng-Hua Li et al. [11] through bioinformatics analysis, also reported that the mRNA levels of SORBS1 was positively correlated with macrophages in bladder cancer, but there were no corresponding basic experiments to confirm this and no in-depth studies were conducted. Therefore, this is the first study to confirm that SORBS1 can regulate macrophage polarization.

The present study is significant and innovative because it is the first systematic analysis of the association between SORBS1 and BRCA, the first report regarding the potential relationship between SORBS1 expression and macrophage polarization, and the first to demonstrate that SORBS1 expression regulates the PI3K/AKT signaling pathway, which in turn affects EMT of breast cancer cells (the SORBS1 action mechanism is detailed in Supplementary Figure 2). However, this study has some limitations. First, since most of our studies were based on bioinformatics analysis and *in vitro* cellular experiments, more *in vivo* experiments need to be designed. Second, we explored the relationship between SORBS1, the PI3K/AKT signaling pathway, and macrophage polarization; however, these conclusions are still immature, and more rigorous inverse experiments need to be designed to verify our conclusions.

## CONCLUSIONS

In conclusion, our findings support that SORBS1 provides important information as a predictive biomarker for BRCA diagnosis. In addition, SORBS1 may inhibit breast cancer cell viability and EMT process by regulating the PI3K-Akt signaling pathway and inducing macrophage polarization.

## Supplementary Material

Supplementary Figures

Supplementary Tables
