# Supplementary material for: SORBS1 inhibits epithelial to mesenchymal transition (EMT) of breast cancer cells by regulating PI3K/AKT signaling and macrophage phenotypic polarization
Source: Aging (Albany NY). 2024 Mar 6;16(5):4789–810. doi: 10.18632/aging.205632 (PMC10968685; doi:10.18632/aging.205632)
Supplement: Supplementary Figures [file aging-16-205632-s001.pdf]

SUPPLEMENTARY FIGURES

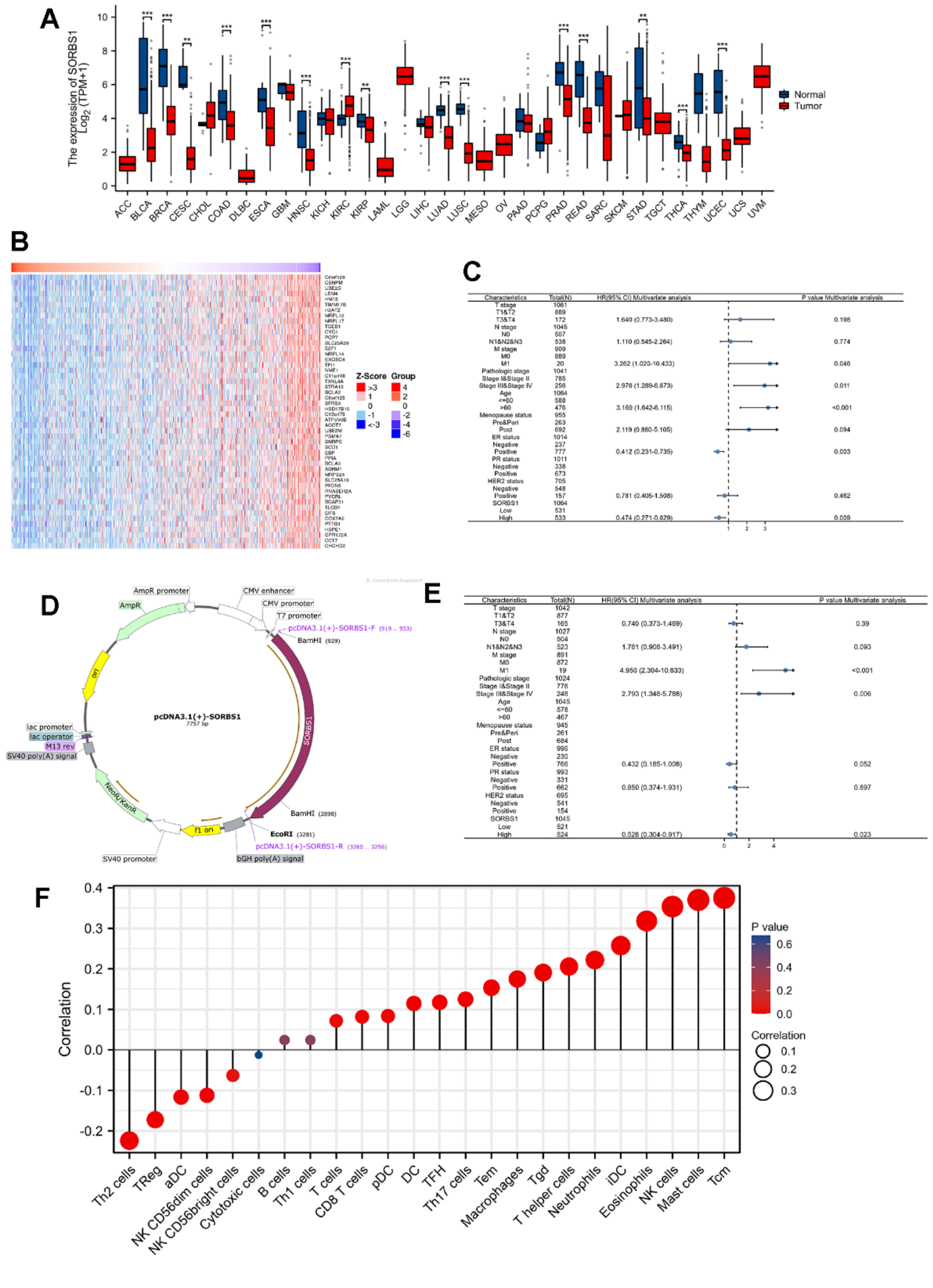

Supplementary Figure 1. Bioassay analysis and mapping of overexpression SORBS1 constructs. (**A**) Expression of SORBS1 in different types of tumors compared with normal tissues in TCGA databases. (**B**) The heat map shows the top 50 genes negatively related to SORBS1 in the BRCA cohort. (**C**) Forest map based on multivariate Cox analysis for overall survival. (**D**) Forest map based on multivariate Cox analysis for disease-specific survival. Analysis for disease-specific survival. (**E**) Construction of the SORBS1 overexpression plasmid map. (**F**) Correlation between SORBS1 expression and relative abundance of 24 types of immune cell. The size of dot corresponds to the absolute Spearman's correlation coefficient values.

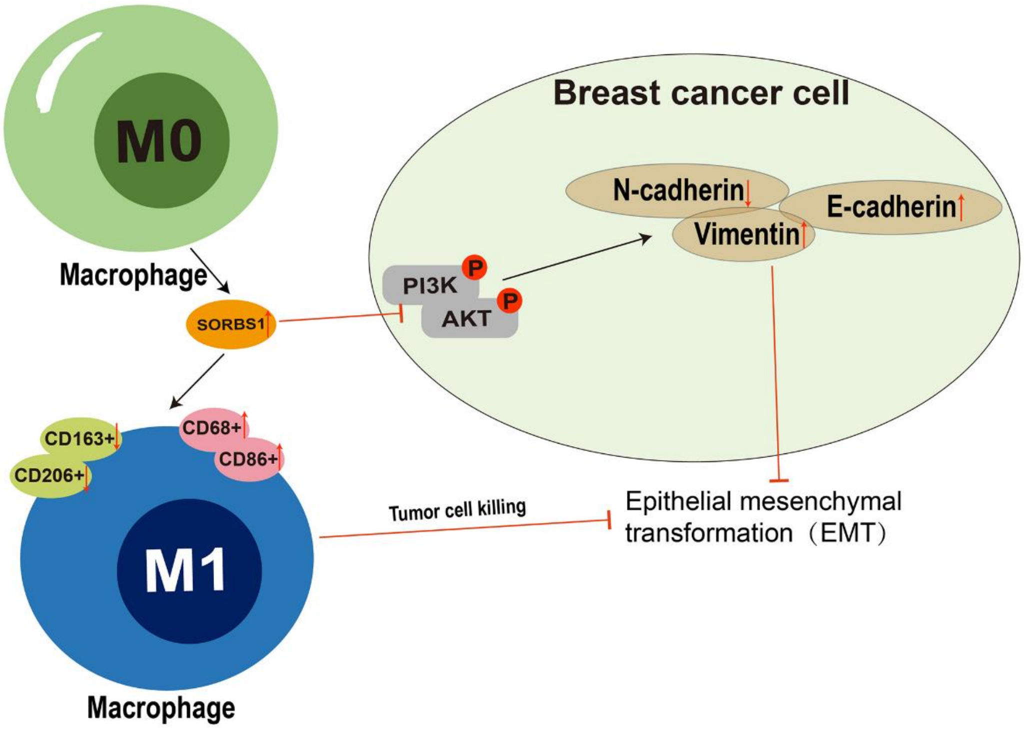

Supplementary Figure 2. A map of the mechanism of SORBS1 action in breast cancer cell lines. SORBS1 inhibits the PI3K/AKT signaling pathway by inhibiting the phosphorylation of PI3K and AKT. Its downstream EMT-related proteins E-cadherin and Vimentin are up-regulated, while N-cadherin is down-regulated, thus blocking the EMT process of breast cancer cells. In addition, overexpression of SORBS1 can also up-regulate the expression levels of CD68 and CD86, and down-regulate the expression levels of CD206 and CD163, thus leading to the differentiation of macrophages to M1 type, enhancing the killing effect on breast cancer cell lines and inhibiting EMT process. (Red lines represent inhibition; The red arrow up indicates increased expression; Red arrow down means decreased expression).
