# Supplementary material for: SORBS1 inhibits epithelial to mesenchymal transition (EMT) of breast cancer cells by regulating PI3K/AKT signaling and macrophage phenotypic polarization
Source: Aging (Albany NY). 2024 Mar 6;16(5):4789–810. doi: 10.18632/aging.205632 (PMC10968685; doi:10.18632/aging.205632)
Supplement: Supplementary Tables [file aging-16-205632-s002.pdf]

SUPPLEMENTARY TABLES

Supplementary Table 1. The clinicopathological characteristics of patients with breast cancer from TCGA.

Characteristic	Levels	Overall
n		1083
T stage, n (%)	T1	277 (25.6%)
	T2	629 (58.2%)
	T3	139 (12.9%)
	T4	35 (3.2%)
N stage, n (%)	N0	514 (48.3%)
	N1	358 (33.6%)
	N2	116 (10.9%)
	N3	76 (7.1%)
M stage, n (%)	M0	902 (97.8%)
	M1	20 (2.2%)
Pathologic stage, n (%)	Stage I	181 (17.1%)
	Stage II	619 (58.4%)
	Stage III	242 (22.8%)
	Stage IV	18 (1.7%)
Race, n (%)	Asian	60 (6%)
	Black or African American	181 (18.2%)
	White	753 (75.8%)
Age, n (%)	<=60	601 (55.5%)
	>60	482 (44.5%)
PR status, n (%)	Negative	342 (33.1%)
	Indeterminate	4 (0.4%)
	Positive	688 (66.5%)
Histological type, n (%)	Infiltrating Ductal Carcinoma	772 (79%)
	Infiltrating Lobular Carcinoma	205 (21%)
ER status, n (%)	Negative	240 (23.2%)
	Indeterminate	2 (0.2%)
	Positive	793 (76.6%)
HER2 status, n (%)	Negative	558 (76.8%)
	Indeterminate	12 (1.7%)
	Positive	157 (21.6%)
PAM50, n (%)	Normal	40 (3.7%)
	LumA	562 (51.9%)
	LumB	204 (18.8%)
	Her2	82 (7.6%)
	Basal	195 (18%)
Menopause status, n (%)	Pre	229 (23.6%)
	Peri	40 (4.1%)
	Post	703 (72.3%)
Anatomic neoplasm subdivisions, n (%)	Left	563 (52%)
	Right	520 (48%)
radiation_therapy, n (%)	No	434 (44%)
	Yes	553 (56%)
OS event, n (%)	Alive	931 (86%)

	Dead	152 (14%)
DSS event, n (%)	Alive	978 (92%)
	Dead	85 (8%)
PFI event, n (%)	Alive	936 (86.4%)
	Dead	147 (13.6%)
Age, median (IQR)		58 (48.5, 67)

Supplementary Table 2. Correlations between OS and mRNA expression of SORBS1 analyzed by univariate and multivariate Cox regression.

Characteristics	Total (N)	Univariate analysis		Multivariate analysis	
		Hazard ratio (95% CI)	P-value	Hazard ratio (95% CI)	P-value
T stage	1061				
T1&T2	889	Reference			
T3&T4	172	1.673 (1.152-2.429)	**0.007**	1.640 (0.773-3.480)	0.198
N stage	1045				
N0	507	Reference			
N1&N2&N3	538	2.145 (1.497-3.073)	**<0.001**	1.110 (0.545-2.264)	0.774
M stage	909				
M0	889	Reference			
M1	20	4.327 (2.508-7.465)	**<0.001**	3.262 (1.020-10.433)	**0.046**
Pathologic stage	1041				
Stage I&Stage II	785	Reference			
Stage III&Stage IV	256	2.519 (1.787-3.549)	**<0.001**	2.976 (1.289-6.873)	**0.011**
Age	1064				
<=60	588	Reference			
>60	476	2.036 (1.468-2.822)	**<0.001**	3.169 (1.642-6.115)	**<0.001**
Menopause status	955				
Pre&Peri	263	Reference			
Post	692	2.405 (1.445-4.002)	**<0.001**	2.119 (0.880-5.105)	0.094
ER status	1014				
Negative	237	Reference			
Positive	777	0.704 (0.487-1.017)	0.062	0.412 (0.231-0.735)	**0.003**
PR status	1011				
Negative	338	Reference			
Positive	673	0.762 (0.541-1.074)	0.120		
HER2 status	705				
Negative	548	Reference			
Positive	157	1.611 (0.981-2.644)	0.059	0.781 (0.405-1.508)	0.462
SORBS1	1064				
Low	531	Reference			
High	533	0.580 (0.414-0.812)	**0.002**	0.474 (0.271-0.829)	**0.009**

Supplementary Table 3. Correlations between DSS and mRNA expression of SORBS1 analyzed by univariate and multivariate Cox regression.

Characteristics	Total (N)	Univariate analysis		Multivariate analysis	
		Hazard ratio (95% CI)	P-value	Hazard ratio (95% CI)	P-value
T stage	1042				
T1&T2	877	Reference			
T3&T4	165	2.037 (1.252-3.314)	**0.004**	0.740 (0.373-1.469)	0.390
N stage	1027				
N0	504	Reference			
N1&N2&N3	523	3.584 (2.089-6.148)	**<0.001**	1.781 (0.908-3.491)	0.093
M stage	891				
M0	872	Reference			
M1	19	7.697 (4.112-14.407)	**<0.001**	4.950 (2.304-10.633)	**<0.001**
Pathologic stage	1024				
Stage I&Stage II	776	Reference			
Stage III&Stage IV	248	3.870 (2.474-6.055)	**<0.001**	2.793 (1.348-5.788)	**0.006**
Age	1045				
<=60	578	Reference			
>60	467	1.418 (0.913-2.201)	0.120		
Menopause status	945				
Pre&Peri	261	Reference			
Post	684	1.591 (0.872-2.904)	0.130		
ER status	996				
Negative	230	Reference			
Positive	766	0.523 (0.326-0.838)	**0.007**	0.432 (0.185-1.008)	0.052
PR status	993				
Negative	331	Reference			
Positive	662	0.529 (0.336-0.833)	**0.006**	0.850 (0.374-1.931)	0.697
HER2 status	695				
Negative	541	Reference			
Positive	154	1.481 (0.740-2.965)	0.267		
SORBS1	1045				
Low	521	Reference			
High	524	0.485 (0.306-0.771)	**0.002**	0.528 (0.304-0.917)	**0.023**

Supplementary Table 4. QPCR primer sequence with siRNA interference sequence information.

Gene name	Forward primer	Reverse primer
SORBS1	CTGGAGGCCACTTGTGGTTC	CCTGGGCATGTCTTGCTTATG-3
CD68	ATGAGGCTGGCTGTGCTTTT	CTAGTGGTGGCAGGACTGTG
CD206	CCAAACGCCTTCATTTGCCA	ACCTTCCTTGCACCCTGATG
β-actin	CTCGCCTTT GCCGATCC	TTCTCCATGTCGTCCCAGTT
Si-SORBS1	GGUACAAGACUAUGUUUAAAC	UUAAACAUAGUCUUGUACCAG
Si-SORBS2	GGUACUUCAAGAAGGACAAAG	UUGUCCUUCUUGAAGUACCAA
Si-SORBS3	CAGAAGAGAUAGAUUUAAAGA	UUUAAAUCUAUCUCUUCUGGG
